# Cerebral malaria: insight into pathology from optical coherence tomography

**DOI:** 10.1038/s41598-021-94495-9

**Published:** 2021-08-03

**Authors:** Zhanhan Tu, Jack Gormley, Viral Sheth, Karl B. Seydel, Terrie Taylor, Nicholas Beare, Valentina Barrera, Frank A. Proudlock, Chatonda Manda, Simon Harding, Irene Gottlob

**Affiliations:** 1 Department of Neuroscience, Psychology and Behaviour, Ulverscroft Eye Unit, University of Leicester, Robert Kilpatrick Clinical Sciences Building, Leicester Royal Infirmary, Leicester, LE2 7LX UK; 2 Department of Eye and Vision Science, Institute of Ageing and Chronic Disease, University of Liverpool, Member of Liverpool Health Partners, Liverpool, UK; 3 Blantyre Malaria Project, University of Malawi College of Medicine, Blantyre, Malawi; 4 Department of Osteopathic Medical Specialties, College of Osteopathic Medicine, Michigan State University, East Lansing, MI USA; 5 University of Malawi College of Medicine, Blantyre, Malawi

**Keywords:** Malaria, Paediatric research, Hypoxia

## Abstract

We aimed to investigate structural retinal changes in malarial retinopathy (MR) using hand-held optical coherence tomography (HH-OCT) to assess its diagnostic potential. Children with MR (n = 43) underwent ophthalmoscopy, fluorescein angiography and HH-OCT during admission, 1-month (n = 31) and 1-year (n = 8) post-discharge. Controls were comatose patients without malaria (n = 6) and age/sex-matched healthy children (n = 43). OCT changes and retinal layer thicknesses were compared. On HH-OCT, hyper-reflective areas (HRAs) were seen in the inner retina of 81% of MR patients, corresponding to ischaemic retinal whitening on fundus photography. Cotton wool spots were present in 37% and abnormal hyper-reflective dots, co-localized to capillary plexus, in 93%. Hyper-reflective vessel walls were present in 84%, and intra-retinal cysts in 9%. Vascular changes and cysts resolved within 48 h. HRAs developed into retinal thinning at 1 month (*p* = 0.027) which was more pronounced after 1 year (*p* = 0.009). Ischaemic retinal whitening is located within inner retinal layers, distinguishing it from cotton wool spots. Vascular hyper-reflectivity may represent the sequestration of parasitized erythrocytes in vessels, a key CM feature. The mechanisms of post-ischemic retinal atrophy and cerebral atrophy with cognitive impairment may be similar in CM survivors. HH-OCT has the potential for monitoring patients, treatment response and predicting neurological deficits.

## Introduction

Cerebral malaria (CM) is a severe complication of *Plasmodium falciparum* infection. In 2019, approximately 229 million episodes resulted in death in 15–25% of patients and neurological morbidity in 30% of survivors^1–4^. The pathogenesis is incompletely understood but intravascular sequestration of parasitized red blood cells (pRBCs) is a central feature. Sequestration occurs in various organs including the brain, leading to vascular congestion^5,6^, impaired perfusion^7^, endothelial cell activation^8,9^, blood–brain barrier breakdown^10^ and cerebral edema^11^, combined with systemic inflammatory responses^12,13^ and a prothrombotic state^14^.

Diagnosis of CM in endemic areas is difficult as asymptomatic parasitemia is common. On autopsy, 23% of children fulfilling WHO criteria for CM^15^ had non-malarial causes of death^5^.

Malarial retinopathy (MR) includes retinal whitening, white-centered hemorrhages and discolored vessels^2,5,16^ and is better than other clinical or laboratory features at distinguishing malarial from non-malarial coma^17^. Severity of MR predicts prolonged coma, neurological sequelae and death^18^. The exact time course of MR is unclear^13^.

Optical coherence tomography (OCT) allows non-invasive visualization and measurement of retinal layers at near-microscopic resolution and has revolutionized the diagnosis and treatment of retinal diseases. Recently, hand-held OCT (HH-OCT), suitable for children and comatose patients, has been developed^19^. HH-OCT has the potential to be a clinical tool in human CM.

Our purpose was to characterize retinal features of CM in vivo using HH-OCT and to evaluate its potential to improve diagnosis and management. We aimed to: (1) characterize OCT changes in MR and compare them to fundus photography, fluorescein angiography (FA), and histological features seen in other CM patients in post-mortem studies; (2) investigate the specificity of OCT features by comparing CM patients to patients in coma due to other causes and healthy controls; (3) compare the thickness of retinal layers in CM patients, control subjects, and clinical parameters; (4) describe longitudinal changes.

## Material and methods

### Study design and ethics

This prospective observational study was performed at Queen Elizabeth Central Hospital (QECH) in Blantyre, Malawi (February 2016 to April 2017). The data collection protocol was approved by the ethical review committee of the University of Malawi, College of Medicine and implemented in accordance with the ethical standards of the 1964 Declaration of Helsinki and its later amendments. As all the participants were younger than 12 years old, informed written consent was obtained from parents/guardians.

### Participants

#### Cerebral malaria

All new cases with the diagnosis of CM admitted to the Pediatric Research Ward were approached. Inclusion criteria were: (1) Blantyre Coma Score ≤ 2 (see Supplementary Table S1^20^); (2) peripheral parasitemia with *P. falciparum;* (3) MR, and (4) no other discernible cause of coma^1^. MR was defined as: (1) retinal hemorrhages; (2) retinal whitening; and/or (3) orange or white retinal vessels seen on fundus examination. Isolated papilledema did not indicate MR^5^.

Standard clinical care and antimalarial treatment (intravenous artesunate) were instituted^20^. In patients whose vital signs were stable, ophthalmoscopy, color photos (Topcon50-EX, Tokyo), FA^21^ were performed at admission. There were 43 children with CM included in this study. HH-OCT scans were repeated daily until discharge to detect the changes during the acute phase, and, in survivors, at one month and one year after discharge when possible.

#### Control participants

There were two groups of control participants recruited:

1. *Comatose children without CM*  To exclude any OCT changes due to coma but not to *P. falciparum* infection, 6 comatose children without CM were included as a control group. According to WHO guidelines, inclusion criteria for comatose children without CM were children with Blantyre Coma Score ≤ 2 but without peripheral parasitemia of *P. falciparum.* Parents/guardians of 6 comatose patients without parasitemia have been approached. They have all agreed to participate in the study.
2. *Healthy children* Healthy local participants with normal visual acuity and fundi were recruited from other pediatric clinics/wards and local communities around hospital, if parents/guardians had sufficient time to participate. They were age- and sex-matched to patients. Children with history of ophthalmological, neurological abnormalities or CM were excluded. Three out of the 46 healthy children were excluded due to poor cooperation.

### Optical coherence tomography (HH-OCT)

Macula HH-OCT scans (Leica Microsystems™ Envisu C-Class, Milton Keynes, UK) of 12 × 8 × 2 mm volume were obtained (600 A-scans and 80 B-scans) (see Supplementary Fig. S1, an example of a control child). Averaging scans is not possible with the hand-held OCT as small movements of the probe or patients will cause artefacts. At admission, pathological changes on HHOCT scans collected from CM patients were compared to fundus pictures (see Supplementary Fig. S1D, an example of a control child) and FAs (see Supplementary Fig. S1E, an example of a control child) taken from the same children. For comparison of the same retinal areas location of retinal vessels was used for the three different image modalities (for methodology see Supplementary Fig. S1, an example of a control child). HH-OCT scans from CM patients were also compared with scans collected from two control groups.

The thickness of the inner and outer retina was measured with manual segmentation (custom macro; http://imagej.nih.gov/ij/; National Institutes of Health, Bethesda, USA) at the foveal center and parafovea between 4° and 6° nasally and temporally (see Supplementary Fig. S1C)^22^. Blurry OCT images where retinal layers could not be clearly identified were excluded and retaken until images with sufficient quality were obtained.

### Statistical methods

Linear mixed models (SPSS, version 24, IBM Analytics) adjusted for age, including eye (right/left), and sex (male/female) as factors, were used to investigate: (1) differences in retinal layer thicknesses between patients and controls; (2) longitudinal changes in thicknesses of retinal layers; (3) correlation with clinical data. Bonferroni post-hoc correction was applied for multiple comparisons.

### Presentations

This study was presented at the 2017 ARVO Annual Meeting (Changes on admission), held in Baltimore, Maryland, May 06–11, 2017/at the 2019 ARVO Annual Meeting (Longitudinal findings), held in Vancouver, British Columbia, Apr 28–May 02, 2019/at the 2018 EPOS meeting, held in Budapest, Hungary, September 7–9, 2018.

## Results

### Characteristics of subjects

Demographic and clinical characteristics of CM patients and healthy controls are shown in Tables 1 and 2, respectively. On admission, OCT scans were collected from 43 patients with CM. Sixty-eight high-quality scans were utilized for quantitative measurements. Forty-three healthy controls were recruited, and 68 OCT images were analyzed to match the ages of the CM patients from whom good quality images were obtained. There were 31 OCT images analyzed at 1-month post discharge and 15 OCT images at 1-year post discharge from CM patients and controls. Six comatose children without CM (12 eyes) were recruited (see Supplementary Table S2). Visual acuities were within the normal age range for all patients at discharge and at follow-up examination and for all healthy control children (0.25DS to + 0.50DS)^23^. None of the participants had cataract and floaters during indirect fundus examinations. We have also checked en-face OCT scans and eliminated OCT scans with artifacts from the analysis.

**Table 1:** Demographic data of patients with cerebral malaria (CM) and controls on admission and follow-up visits.

Groups	Number of participants	Age (months)		Number of males	OCT images (eyes)			Fluorescein angiography (eyes)
		Range	Mean		On admission	24 h after admission	48 h after admission	
**Cerebral malaria patients on admission**								
CM patients	43	7–131	55	21	86 (68 high quality)	84	48	64
Healthy controls	43	9–138	56	21	68 analyzed	··	··	··

Groups	Number of patients	Age (months)		Number of males	OCT images (eyes)			··
		Range	Mean		Total scans	With HRAs	Without HRAs	
**Cerebral malaria patients at 1 month follow-up**								
CM patients	18	12–96	54	8	36 (31 high quality)	18	13	··
Healthy controls	18	11–107	55	8	31 analyzed	··	··	··
**Cerebral malaria patients at 1 year follow-up**								
CM patients	8	26–140	73	4	16 (15 high quality)	13	2	··
Healthy controls	8	26–140	73	4	15 analyzed	··	··	··

**Table 2:** Clinical data in CM patients.

Age (months)	54 ± 30^§^, age range 7–131
Male sex—no. (%)	21 (49)
**Variable before admission**	
Duration of fever (h)	56 ± 32^§^
Duration of coma (h)	13 ± 12^§^
**Variable on admission**	
Temperature (°C)	39 ± 1^§^
Blantyre coma score (%)	
0	2
1	13
2	18
Packed cell volume	23 ± 5.9^§^
CSF opening pressure (mm of water)	
Median	170
Interquartile range	115–240
Parasitemia (parasites/mm^3^)	
Median	4697
Interquartile range	189–53,520
HRP2 (ng/ml)	
Median	1082
Interquartile range	562–3177
White cells (× 10^−9^/l)	
Median	8
Interquartile range	6.4–11.45
Platelets (× 10^−9^/l)	
Median	64
Interquartile range	43.3–88
Hypoglycemia—no. (%)	0 (0)
Hyperlactatemia—no. (%)	15 (35)
**Discharge outcomes**	
Coma resolution time (h)	36 ± 28^§^
Fever clearance time (h)	38 ± 42^§^
Parasite clearance time (h)	26 ± 10^§^
Outcomes—death (%)	1 (2)

Data with ‘^§^’ presented as mean ± SD.

*HRP2* histidine-rich protein 2 concentration, *CSF* cerebrospinal fluid opening pressure, *Hyperlactatemia* an increased level of lactate in the blood (> 5 mmol/l), *Hypoglycaemia* the glucose (blood sugar) level lower than 3 mmol/l, *Coma resolution time* the time from admission to recovery reaching and sustaining coma score 5.

### OCT findings in children with MR on admission

#### Hyper-reflective capillaries

Abnormal hyper-reflective well-defined dots located in the parafoveal region were seen on OCT scans in 93% of CM eyes (Fig. 1.1.A) in the area corresponding to capillary beds of the superficial, intermediate and deep retinal plexuses (green, blue and orange arrows respectively) (Fig. 1.1.A,E^24^). Histology of MR from a different patient^25^ not included in the OCT study (Fig. 1.1.D) shows parasite sequestration in capillaries and venules (black arrows—high sequestration *vs.* black arrowheads—low sequestration) in similar locations as hyper-reflective dots. Affected capillaries were not visible on en-face OCT (Fig. 1.1.B) and color photograph (Fig. 1.1.C) and occurred with corresponding capillary non-perfusion seen on FA (Fig. 1.1.F). To show the differences between the abnormal hyper-reflective vessels and normal vessels in a healthy control on a B-scan, a side-by-side comparison is shown in Supplementary Fig. S2A (CM patient) and S2B (healthy control).

**Figure 1:**
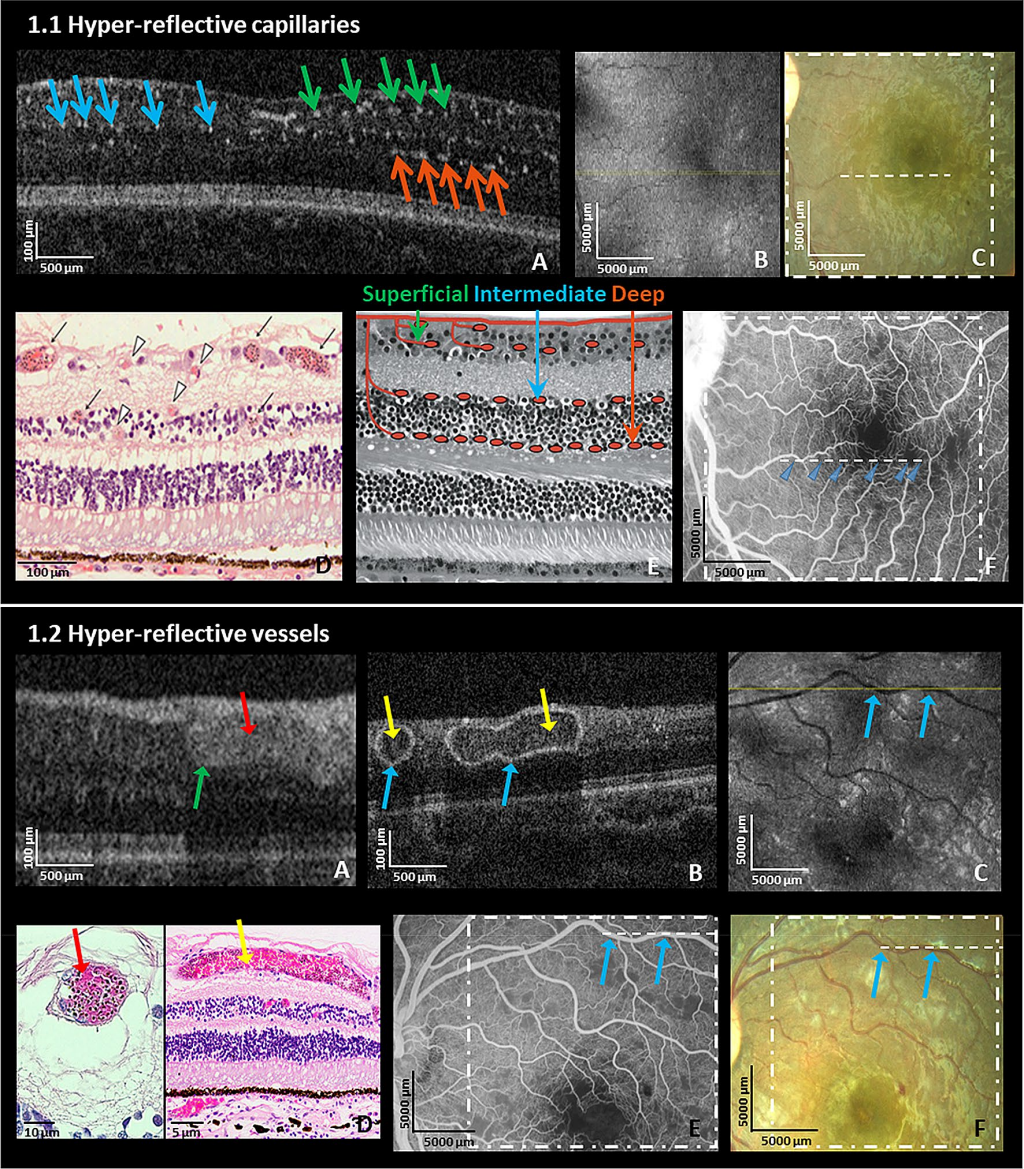
Hyper-reflective capillaries and vessels. (**1.1**) Left eye of a 7-month old male CM patient on admission. (**A**) HH-OCT B-scan showing hyper-reflective dots corresponding to locations of capillaries (green, blue and orange arrows: superficial, intermediate and deep capillary plexus); (**B**) En-face OCT displays no visible change; (yellow line: location of the OCT B-scan in the image (**A**)). (**C**) Fundus photo; dashed line and square correspond to (**A, B**); (**D**) Representative retinal cross-section histology from a different fatal patient showing capillaries and venules affected by parasite sequestration (black arrows: intense sequestration; arrowheads: low or no sequestration); Reprinted with permission^25^; (**E**) Schematic representation of superficial, intermediate and deep capillary plexus collocated to capillaries filled with pRBCs on OCT in (**A**). Reprinted with permission^24^; (**F**) Fundus fluorescein angiography (FA) shows perfusion deficits of capillaries (blue arrows) corresponding to parasitized vessels on OCT in the same location of (**1.1.A**). (**1.2**) Left eye of a 42-month old male CM patient on admission. (**A**) Hyper-reflective vessel (blue arrow) corresponding to localization of vessel on fundus photo and FA (not shown) with hyper-reflective lumen (red arrow); and (**B**) Hyper-reflective vessels (blue arrows) with hypo-reflective lumina (yellow arrows) on OCT B-scans; (**C**) En-face OCT (yellow line: location of OCT B-scan in B, blue arrows: vessels shown in (**B**); (**D**) Representative retinal cross-section histology showing pRBCs in venules from a different fatal patient (red arrow shows vessels with pRBCs filling the lumen; yellow arrow shows a vessel where pRBCs cytoadhere to the vessel wall with red blood cells without parasites in the center of the vessel (similar to hypo-reflective lumina of blood vessels in (**1.2.B**); (**E**) Fundus fluorescein angiography and (**F**) Fundus photo corresponding to OCT in (**1.2.B**) (blue arrows corresponding to vessels in the image (**B**); dashed line and square correspond to (**B, C**).

#### Hyper-reflective vessels

Hyper-reflective ring-like or ovoid structures (Fig. 1.2.A, B) corresponding to the location of retinal vessels on en-face images (Fig. 1.2.C), were seen on OCT in 90% of eyes. The walls of the veins, arteries, venules and arterioles were hyper-reflective on OCT (Fig. 1.2.A, B). The lumina were hyper-reflective (84%) (Fig. 1.2.A; red arrow) or hypo-reflective (73%) (Fig. 1.2.B; yellow arrow). In 67% of eyes, both were present simultaneously. Figure 1.2.D (red arrow) represents histology from a different patient not included in the OCT study shows a vessel where both wall and lumen are filled with parasitized red blood cells (pRBCs), comparable to vessels on OCT with hyper-reflective lumina (example in Fig. 1.2.A (red arrow)). Figure 1.2.D (yellow arrow) shows dense sequestration of erythrocytes on the vessel wall with preservation of the blood flow in the hypo-reflective lumen comparable to OCT with hypo-reflective lumen (Fig. 1.2.B yellow arrow). Hyper-reflective vessels looked predominantly normal on FA (Fig. 1.2.E) and funduscopy (Fig. 1.2.F). Side-by-side comparisons of vascular walls of a b-scan of a normal healthy age-and gender-matched eye (see Supplementary Fig. S2D) is shown for comparison with hyper-reflective vessels in patients with CM (see Supplementary Fig. S2C).

#### Hyper-reflective areas (HRAs)

Hyper-reflective areas (HRAs) with variable size and diffuse borders, appeared as high reflectance in the inner nuclear layer (INL), outer plexiform layer (OPL) and the outer nuclear layer (ONL) (Fig. 2.1.A; white circle and B, white arrows) in 81% of eyes. Larger HRAs corresponded to dark areas on the en-face image (Fig. 2.1.C; white arrows). They were located predominantly in the parafoveal area with a propensity for the temporal raphe (watershed zone) and spared the foveolae. Histology from the literature^17^ shows immunochemical staining of fibrinogen leaking from vessels into the OPL (Fig. 2.1.D black arrowhead) suggestive of a breakdown of the blood-retinal barrier at similar locations and distributions than HRAs. Larger HRAs were visible as capillary non-perfusion on FA (Fig. 2.1.E; white arrows), and whitening on fundus photos (Fig. 2.1.F; white arrows) while small HRAs were not always apparent^26^.

**Figure 2:**
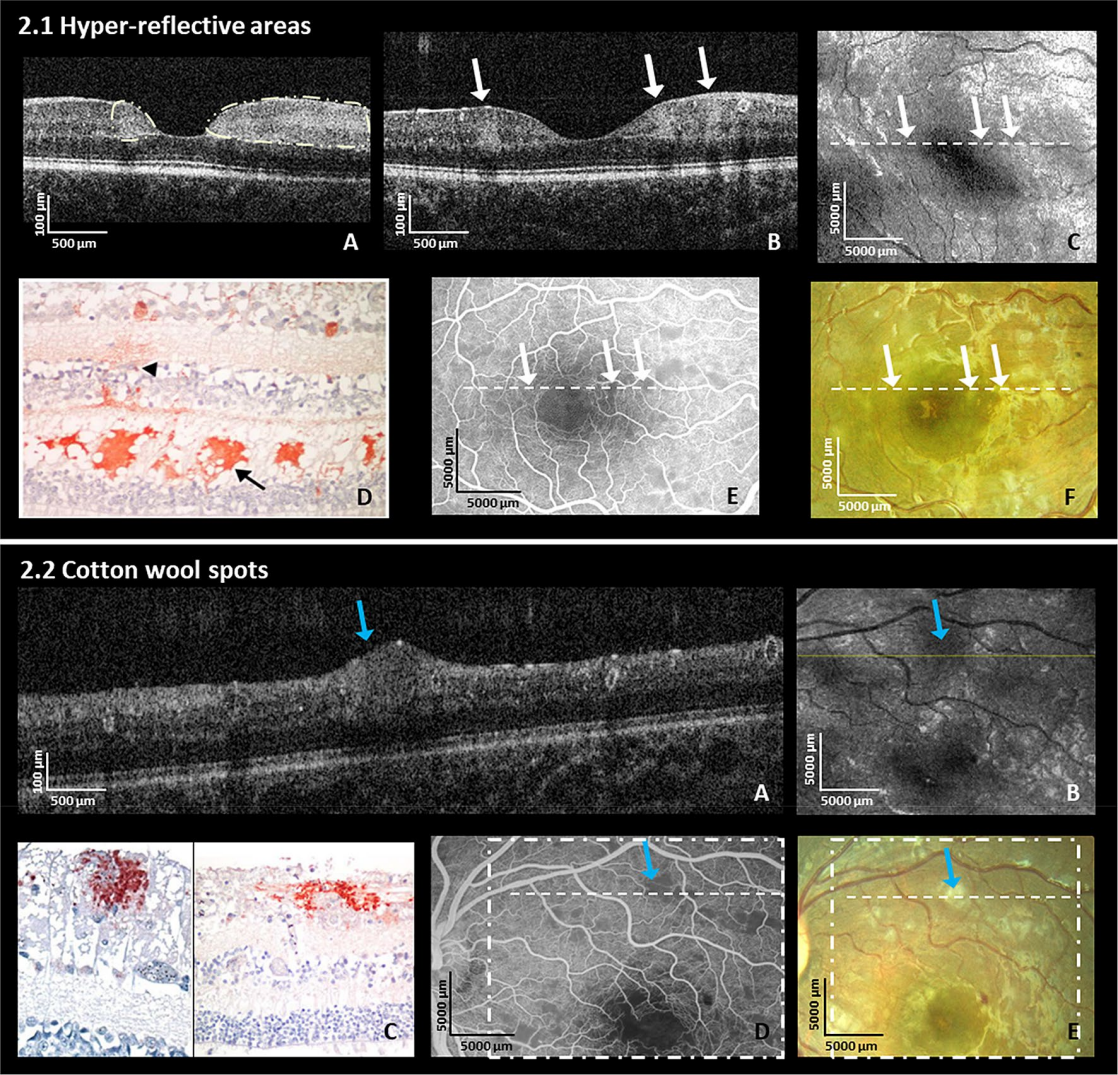
Hyper-reflective areas and cotton wool spots (Retinal Whitening). (**2.1.A, B**) shows multiple hyper-reflective areas of various sizes (white arrows) located in the inner nuclear layer level, the outer plexiform layer and outer nuclear layer on OCT B-scans in the right eye of a 22-month old male CM patient and in the left eye of an 84-month old female CM patient at admission, respectively; (**C**) En-face OCT of the patient in (**B**); white arrows correspond to the location of white arrows in the image (**B**) (hyper-reflective areas); The hyper-reflective areas in OCT B-scan (**B**) show darkening on the en-face image (**C**) in this patient (white arrows); (**D**) Representative histology of immuno-histochemical staining for fibrinogen surrounding a small vessel (black arrowhead) mainly located in OPL (black arrow) from the literature (Reprinted with permission)^17^. (**E**) Fundus fluorescein angiography showing hypo-perfusion of capillaries and (**F**) fundus photo same eye as OCT in (**B**); white arrows and dashed lines show parafoveal whitening which are corresponding to the same fundus locations in (**B, C, E, F**). (**2.2.A**) Cotton wool spot (blue arrow) in the left eye at admission on OCT B-scan in the left eye of a 42-months old male CM patient at admission; (**B**) En-face OCT with darkening in area of cotton wool spot (blue arrow); fine yellow line: location of the B-scan in the image (**A**); blue arrow corresponds to blue arrow in image (**A**); (**C**) Immunohistochemical staining for b-APP in retinal nerve fiber layer (RNFL) from different CM patients. Reprinted with permission^17^. (**D**) Fundus fluorescein angiography showing grey area masking underlying capillaries (blue arrow) and (**E**) fundus photo from patient’s left eye corresponding to OCT showing white cotton wool spot (blue arrow) in (**2.2.A**). The dashed square and the dashed line correspond to en-face OCT in B and B-scan in (**A**). Blue arrows show the location of a cotton wool spot. Blue arrows correspond to the same fundus locations in (**A, B, D, E**).

#### Cotton wool spots (CWS) (Fig. 2.2)

CWS were distinct from HRAs on OCT, being located in the retinal nerve fiber layer (RNFL), raised and well circumscribed. CWS distorted inner and outer retinal layers with decreased signal density compared to HRAs (Fig. 2.2.A) (37% of eyes). They were less straightforward to distinguish from retinal whitening on color photos as white/grey feathered spots (Fig. 2.2.E)^26^. On the en-face OCT, CWS had a dark appearance (Fig. 2.2.B). Histology from a different patient^17^ not included in the OCT study (Fig. 2.2.C) shows b-APP staining in the RNFL indicative of axonal damage located in the same retinal layers as CWS. On FA CWS corresponded to grey areas masking underlying capillaries (Fig. 2.2.D).

#### White-centered hemorrhage (see Supplementary Fig. S3.1)

White-centered hemorrhages corresponded to superficial well-circumscribed lesions below the ILM, or deeper diffuse hyper-reflective lesions involving the inner retina. En-face OCT images show superficial hemorrhages as dark spots or rings matching hemorrhages on color images. Deep white-centered hemorrhages are poorly defined on en-face imaging.

#### Cystoid macular edema (CME) (see Supplementary Fig. S3.2)

CME was present on OCT in four patients (9%) with and without sub-retinal fluid. Cysts were in the OPL of the foveal and parafoveal region: none was detected fundoscopically in any patients.

The frequencies of the various OCT characteristics in patients with MR are summarized in Supplementary Table S3.

## OCT findings in control children

None of the healthy or comatose control children had any retinal abnormalities on OCT.

## Quantitative analysis

## Macular and foveal thickness

On admission, no difference in retinal thickness at the foveal center and parafovea between 4° and 6° nasally and temporally was found between CM patients and controls, although there was a trend to statistical significance for thicker inner retinal layers temporally in the CM group (*p* = 0.06).

Coma resolution time was positively correlated with the thickness of outer retinal layers of temporal macula (*p* = 0.0036) on admission. Thicker outer retinal layers were found in children with longer coma resolution time. No correlations between thicknesses of retinal layers and other clinical parameters were found.

## Longitudinal findings

**Figure 3:**
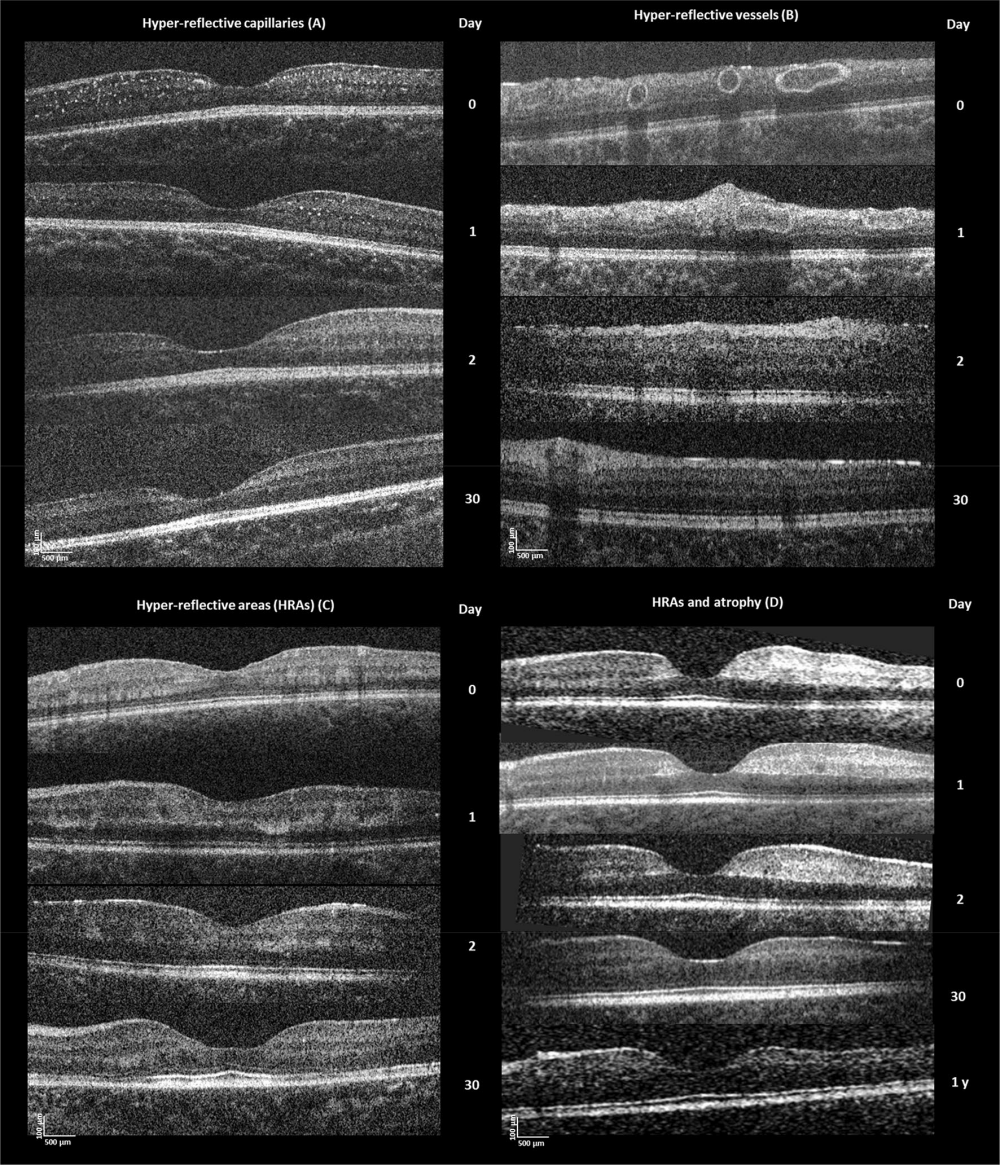
Longitudinal changes in the retina. (**A**) Hyper-reflective capillaries: OCT of a 7-month-old male CM patient at admission, 1 day, 2 days and 30 days. The number of hyper-reflective capillaries diminished rapidly 1 day after treatment initiation; hyper-reflective capillaries were almost not detectable on OCT after 2 days. (**B**) Hyper-reflective vessels: OCT of 42-month-old male CM patient at admission, 1 day, 2 days and 30 days after start of treatment. The abnormal hyper-reflective vessels also diminished rapidly within 24 h. (**C**) Hyper-reflective areas: OCT of 45-month-old female CM patient at admission, 1 day, 2 days and 30 days after start of treatment. The hyper-reflective areas remained visible, but were smaller and less intense at 1-month follow-up. Retinal atrophy was just evident at this time point. (**D**) Parafoveal hyper-reflective areas and atrophy: OCT of 22-month-old male CM patient at admission, 1 day, 2 days, 30 days and 1 year. He had relatively large and dense hyper-reflective areas at admission (particularly to the temporal side of the fovea (right)). There is corresponding retinal atrophy at 1-year follow-up with marked thinning compared to the left of the fovea.

*Vascular changes* Twenty-four hours after starting treatment, the intensity of the hyper-reflective capillaries and vessels diminished on OCT, returning to normal by 48 h in all patients (Fig. 3).

*Hyper-reflective areas (HRAs) and retinal atrophy* HRAs were still visible 1-month post-discharge in 18 eyes (Fig. 3C). Seven out of eight patients who had 1-year follow-up had HRAs at admission, of which five showed visible retinal atrophy corresponding to the previous location of HRAs (Fig. 3D). Most of the volume loss occurred in the inner retina temporally, consistent with the anatomical location of HRAs. The patient without HRAs had no noticeable retinal thinning at 1-year.

## Longitudinal quantitative retinal thickness

*Comparison of retinal layer thickness between admission and follow-up in CM* Compared to admission, significant thinning of inner retinal layers (*p* < 0.0001) was observed at one-month for the entire CM cohort (31 eyes; and patients; 18 eyes with and 13 eyes with and without HRAs) (see Supplementary Fig. S4A). No differences in the outer retinal thickness were observed, except at the foveola where a significant increase of the outer retinal layers was observed (*p* < 0.001). At 1-year follow-up (15 eyes), the thickness of the inner layers had significantly decreased at the temporal parafovea compared to admission (*p* < 0.001) (see Supplementary Fig. S4B).

As retinal atrophy was observed in locations corresponding to HRAs, CM patients with and without HRAs were compared. Significant thinning was found at 1-month (*p* = 0.024) and 1-year (*p* = 0.0009) in patients with HRAs on admission, but not in patients who did not have evidence of HRAs on admission (see Supplementary Fig. S4). As only two eyes without HRAs were scanned at 1-year, statistical analysis was not performed.

**Figure 4:**
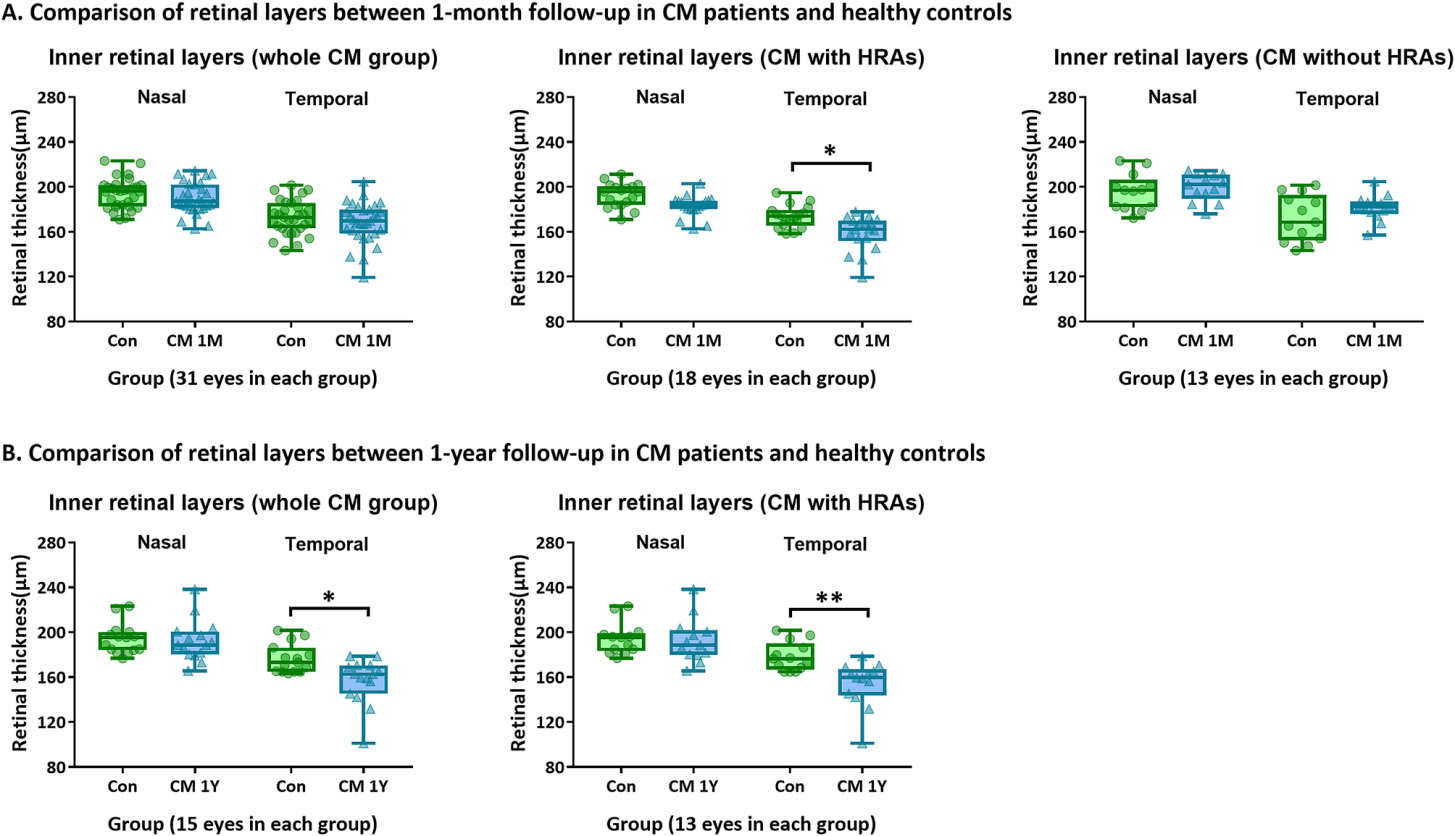
Comparisons of thicknesses of retinal layers between follow-up of CM patients with HRAs and healthy controls. (**A**) Comparison of thicknesses of inner retinal layers between controls and CM patients at 1-month follow-up. (**B**) Comparisons of thicknesses of inner retinal layers between controls and CM patients at 1-year follow-up. Error bars in the boxplot are the ranges of data. *p < 0.05 and **p < 0.01 show significant differences between the two groups. Control groups are in green and patient groups in blue (*Con* controls, *CM* cerebral malaria, *1 M* 1-month follow-up, *1Y* 1-year follow-up).

*Comparison of retinal layers thickness between healthy controls and CM patients at follow-up* At 1-month, no difference was found in the thickness of retinal layers between CM patients and controls. However, when analyzed separately, patients with HRAs at admission (n = 18) had thinning in the inner layers at 1-month, especially on the parafoveal temporal side (*p* = 0.027), while no significant change was found in those without HRAs (n = 13) at admission (Fig. 4A).

At 1-year the CM patients had significant temporal parafoveal thinning compared to controls (*p* = 0.024) (13 eyes with HRAs at admission, *p* = 0.009) (Fig. 4B).

No significant difference was found in outer retinal layers or RNFLs at follow-up.

## Discussion

### Hyper-reflective capillaries and vessels

Hyper-reflective rings or ovoid structures seen in the OCTs of CM patients clearly correspond to retinal vessels, and it is probable that the hyper-reflective walls represent endothelial sequestration of pRBCs, the histological hallmark of CM.

Small hyper-reflective dots are co-localized with retinal capillary plexus, strongly suggesting that they correspond to retinal capillaries affected by sequestration. Our hypothesis is that the hyper-reflectivity may be directly due to the presence of sequestered pRBCs or to a change exerted on the vessel wall. Their rapid resolution with treatment suggests a reversibility which mirrors parasite clearance.

A recent investigation of malarial retinopathy used a mouse model of cerebral malaria to compare changes in OCT to fundus imaging with labelled parasites and histology with immunological markers^27^. It localized the parasites in larger vessels by detecting labelled GFP and in three capillaries layers with CF8 staining, confirming OCT hyper-reflectivity in vessels with sequestration and supporting our conclusions. The locations and morphology of the hyper-reflectivity are consistent with our findings localizing the parasites by detecting GFP expression of clusters of plasmodia in big vessels and all three capillary layers with CF8 staining^27^.

Vessels with hyper-reflective lumens on OCT likely represent lumens filled with pRBCs, as uninfected RBCs have a low OCT signal. A hypo-reflective center in a hyper-reflective lumen is probably a narrow area of central patency. These findings are consistent with the retinal histology in CM^16^. Hyper-reflectivity seen in retinal capillaries and vessels is most likely due to hemozoin, a metabolic by-product of the falciparum parasite^28^. Hemozoin is birefringent on examination with polarised light^28^. Retinal intravascular material seen in retinal vessels on FA indicates sequestration, and is present in the majority of MR positive patients (post-capillary 98.3% and small venules 87.9%)^16^. This is very similar to our OCT data (93% hyper-reflective capillaries and 90% hyper-reflective vessels) further supporting sequestration as the basis of the OCT findings. Vascular changes in the macula on OCT were rarely seen on funduscopy, likely representing the higher resolution and sensitivity of OCT. Vessel abnormalities are more visible in the peripheral retina on clinical examination.

Small hyper-reflective foci have also been found in other inflammatory retinal diseases such as diabetic retinopathy^29^. However, these foci are also located in the outer retinal layers unlike the hyper-reflective dots we found in CM (see Supplementary Fig. S5). Therefore, the hyper-reflective foci in diabetic retinopathy do not follow the distribution of vessels in contrast of the hyper-reflective dots in CM.

Typically, after 48 h of antimalarial treatment, retinal vessels and capillaries appeared normal on OCT. This time course was similar to peripheral blood clearance times (Table 2). Therefore, OCT has the potential to non-invasively assess response to treatment and track the reversal of sequestration in a physiologically relevant setting, a CNS microvascular bed.

### Retinal whitening and hyper-reflective areas

Retinal whitening in MR corresponds to capillary non-perfusion with good evidence it is caused by ischemia^16^ which, at a cellular level may represent cytotoxic edema or oncotic cell swelling due to reduced capillary perfusion^30^, metabolic steal by sequestered parasites^22^ or microthrombi^17^. Immunochemical staining of fibrinogen leaking from vessels suggests that blood-retinal barrier breakdown occurs at similar locations and distributions as HRAs^17^. The co-location of larger HRAs to retinal whitening strongly suggests HRAs are a manifestation of tissue hypoxia.

Our group has previously shown an association between severe retinal whitening with reduced electroretinography b-wave amplitudes, indicating reduced function of the inner nuclear layer. This is consistent with the involvement of the INL in HRAs^31^. CWS are a recognized feature of MR, but can be difficult to differentiate from retinal whitening clinically and on photographs. OCT images are able to differentiate these retinal features more readily^21^. Retinal whitening, mainly located parafoveally, appears to correspond to HRAs at the interface of the INL, OPL, and ONL; by contrast, CWS, located predominantly in the peripheral macula corresponded to hyper-reflective RNFL lesions.

Similar hyper-reflective lesions co-localized with retinal whitening have been described in paracentral acute middle maculopathy (PAMM), an uncommon condition seen in patients with retinal ischemia due to various aetiologies (See Supplementary Fig. S6)^32,33^. As in MR, the foveola is not affected as it is avascular and mainly supplied by choroidal blood flow. HRAs in PAMM and MR appear to be a feature of ischemia^34^. CWS are also whitish lesions, but with OCT are readily distinguishable from retinal whitening. CWS occur due to the disruption of axonal transport in the RNFL. In MR, CWS tend to occur in the peripheral macula and just outside the vascular arcades, and likely represent a border between ischemic and better perfused retina^35^.

### White centered hemorrhage and cystoid macula edema (CME)

On OCT hemorrhages were hyper-reflective and either superficial and well-circumscribed, or deep and diffuse involving the inner retina likely reflecting hemorrhages from superficial and deep capillary plexus. White-centered hemorrhages are typical in MR and are associated histologically with fibrin thrombi (see Supplementary Fig. S3.1.C). Central thrombi may be just discernible in deep hemorrhages on OCT.

CME is difficult to visualize and diagnose using ophthalmoscopy and has probably been missed in studies relying on ophthalmoscopy. It is easily visible on OCT and we found it in nearly 10% of patients (See Supplementary Fig. S3.2.A). CME occurred less frequent in our patients, than in autopsy cases (50%)^17^ which may be explained by the increased severity of disease on cases going on to autopsy. Larger numbers are needed to test whether CME is associated with fatal outcomes.

### Atrophy of inner retinal layers

Inner retinal layers started to thin at 1-month follow-up and by 1-year were clearly atrophic compared to admission, and to controls. Atrophy was associated with the presence of HRAs on admission, indicating the neuronal consequences of acute patchy ischemia on the CNS, and providing a mechanism for the cognitive impairment associated with brain atrophy in CM survivors^4^. Comparison between retinal atrophy on OCT and brain atrophy on MRI as well as cognitive functions are necessary to elucidate to which extent they are associated.

### Correlation of size of retinal layers with clinical parameters

Thicker outer layers were associated with longer coma resolution time, possibly indicating that edema of temporal outer retinal layers is associated with disease severity.

### Limitations of the study

This is a novel study of new HH-OCT technology. Further work will be required to establish the role of HH-OCT from the promising potential we have identified, particularly with regard to the diagnosis of CM, tracking sequestration, and predicting cognitive sequelae.

OCT mainly images the macula within the vascular arcades, while MR changes occur throughout the fundus. However, most MR features occur within the macula, and the higher resolution of OCT and ability to cross-section the retina enables it to detect previously undetected retinal effects.

We only had a small sample size for the 1-year follow-up, but the findings were statistically significant. We also had a small group with coma of non-malarial causes (n = 6). However, none of the larger group of healthy children had any of the MR signs seen on OCT.

Our interpretation of the pathological findings on OCT can only be, to some extent, speculative. This is because a direct comparison of the same cut of an OCT image and postmortem histology is impossible. It is difficult to obtain post-mortem pathology from the same patients from which we obtained OCT as the minority of patients are dying. In addition, retinal pathology changes over time with the course of the disease. For example, twenty-four hours after starting treatment, the intensity of the hyper-reflective capillaries and vessels diminished significantly on OCT, returning to normal by 48 h in all patients (see longitudinal changes in “Results” section, Fig. 1). Therefore, even if we would obtain an eye of a deceased patient from whom we have previously obtained an OCT scan the pathological sample and the OCT scan would be obtained at a different stage of the disease and not reflect the same pathology. It would also not be possible to obtain exactly the same section on postmortem histology and on OCT scan for direct comparison.

OCT angiography (OCTA) would ideally show perfusion deficits in the different retinal plexuses to compare localisation with hyper-reflective dots on OCT b-scans. However, OCTA cannot prove the changes within the vessels and in the retinal tissues such as hyper-reflective zones corresponding to PAMM in the inner retinal layers.

Also, paediatric OCT angiography (OCTA) is not available and OCTA requires stable fixation for at least 27 s for scanning a similar area on the OCT B-scans^36^. The hand-held OCT data are collected in very difficult circumstances on the malaria ward on comatose children. Examiners need to constantly adapt the position of the OCT probe to the position of the patient’s body and eyes flexibly to obtain OCT b-scans. One b-scan takes only 0.025 s. It is not possible to fix the OCT probe for a longer duration during this examination to obtain OCTA. Examiners need to constantly adapt the position of the OCT probe to the position of the patient’s position, as they make involuntary body and eye movements. Although there are limitations in our study, we have used the best dataset in an endemic setting and the best available knowledge comparing the pathology to conventional fluorescein angiography (see Figs. 1, 2, Supplementary Fig. S3) and post-mortem pathology from other patients carefully to reach the current hypothesis. Although our interpretation of data is, to some extent, speculative our paper presents important new findings which could serve as a biomarker for cerebral malaria.

## Conclusion

Our study using non-invasive OCT imaging of the retina has generated new insights into the pathophysiology of MR and CM. The novel finding of hyper-reflective abnormalities in capillaries and vessels most likely corresponds to sequestered pRBCs within these vessels. The rapid disappearance of this hyper-reflectivity indicates that OCT may allow monitoring the effectiveness of treatment on pRBCs sequestration and clearance in vivo. OCT clearly differentiated retinal whitening (HRA affecting the inner retinal layers), from cotton wool spots (affecting the RNFL). HRAs are more common than visible retinal whitening from ischemia. Follow-up data indicated that HRAs lead to retinal atrophy, suggesting a mechanism for cerebral atrophy and high rates of cognitive and developmental impairment after CM.

OCT changes are specific to CM patients with MR, and allow differentiation between malarial and non-malarial coma. We have shown that HH-OCT is a fast, non-invasive method to image neural tissue, especially for young children, its microvasculature and how they are affected by CM in humans, both acutely and in the long term. It can provide insights into pathophysiology but also potentially for better clinical care.

## Supplementary Information

Supplementary Information.

## References

[1] Seydel KB (2015). Brain swelling and death in children with cerebral malaria. N. Engl. J. Med..

[2] Postels DG, Chimalizeni YF, Mallewa M, Boivin MJ, Seydel KB (2013). Pediatric cerebral malaria: A scourge of Africa. Future Neurol..

[3] WHO (2020). World Malaria Report 2020.

[4] Langfitt JT (2019). Neurodevelopmental impairments 1 year after cerebral malaria. Pediatrics.

[5] Taylor TE (2004). Differentiating the pathologies of cerebral malaria by postmortem parasite counts. Nat. Med..

[6] MacPherson GG, Warrell MJ, White NJ, Looareesuwan S, Warrell DA (1985). Human cerebral malaria—A quantitative ultrastructural analysis of parasitized erythrocyte sequestration. Am. J. Pathol..

[7] White N (1985). Pathophysiological and prognostic significance of cerebrospinal-fluid lactate in cerebral malaria. The Lancet.

[8] Turner GDH (1998). Systemic endothelial activation occurs in both mild and severe malaria. Correlating dermal microvascular endothelial cell phenotype and soluble cell adhesion molecules with disease severity. Am. J. Pathol..

[9] Elhassan IM (1994). Evidence of endothelial inflammation, T cell activation, and T cell reallocation in uncomplicated *Plasmodium falciparum* Malaria. Am. J. Trop. Med. Hyg..

[10] Brown (1999). Evidence of blood–brain barrier dysfunction in human cerebral malaria. Neuropathol. Appl. Neurobiol..

[11] Newton CRJC (1997). Intracranial hypertension in Africans with cerebral malaria. Arch. Dis. Child..

[12] Clark IA, Rockett RA, Cowden WB (1993). TNF in cerebral malaria. QJM.

[13] Clark IA, Cowden WB, Rockett KA (1995). Nitric oxide in cerebral malaria. J. Infect. Dis..

[14] Clemens R (1994). Activation of the coagulation cascade in severe falciparum malaria through the intrinsic pathway. Br. J. Haematol..

[15] WHO (2014). Tropical Medicine & International Health.

[16] Barrera V (2018). Neurovascular sequestration in paediatric *P. falciparum* malaria is visible clinically in the retina. Elife.

[17] White VA, Lewallen S, Beare NA, Molyneux ME, Taylor TE (2009). Retinal pathology of pediatric cerebral malaria in Malawi. PLoS ONE.

[18] Beare NA (2004). Prognostic significance and course of retinopathy in children with severe malaria. Arch. Ophthalmol..

[19] Maldonado RS (2010). Optimizing hand-held spectral domain optical coherence tomography imaging for neonates, infants, and children. Investig. Ophthalmol. Vis. Sci..

[20] Molyneux ME, Taylor TE, Wirima JJ, Borgsteinj A (1989). Clinical features and prognostic indicators in paediatric cerebral malaria: A study of 131 Comatose Malawian Children. QJM Int. J. Med..

[21] MacCormick IJ (2015). Grading fluorescein angiograms in malarial retinopathy. Malar J..

[22] Hero M (1997). Photographic and angiographic characterization of the retina of kenyan children with severe malaria. Arch. Ophthalmol..

[23] Sorsby A, Sheridan M, Leary GA, Benjamin B (1960). Vision, visual acuity, and ocular refraction of young men: Findings in a sample of 1,033 subjects. BMJ.

[24] Rahimy E, Kuehlewein L, Sadda SR, Sarraf D (2015). Paracentral acute middle maculopathy: What we knew then and what we know now. Retina (Philadelphia).

[25] Barrera V (2015). Severity of retinopathy parallels the degree of parasite sequestration in the eyes and brains of Malawian children with fatal cerebral malaria. J. Infect. Dis..

[26] Harding SP (2006). Classifying and grading retinal signs in severe malaria. Trop. Doct..

[27] Paquet-Durand F (2019). A retinal model of cerebral malaria. Sci. Rep..

[28] Lawrence C, Olson JA (1986). Birefringent hemozoin identifies malaria. Am. J. Clin. Pathol..

[29] Bolz M (2009). Optical coherence tomographic hyperreflective foci: A morphologic sign of lipid extravasation in diabeticmacular edema. Ophthalmology.

[30] Beare NA, Harding SP, Taylor TE, Lewallen S, Molyneux ME (2009). Perfusion abnormalities in children with cerebral malaria and malarial retinopathy. J. Infect. Dis..

[31] Lochhead J (2010). The effects of hypoxia on the ERG in paediatric cerebral malaria. Eye (Lond.).

[32] Sarraf D (2013). Paracentral acute middle maculopathy: A new variant of acute macular neuroretinopathy associated with retinal capillary ischemia. JAMA Ophthalmol..

[33] Casalino G, Williams M, McAvoy C, Bandello F, Chakravarthy U (2016). Optical coherence tomography angiography in paracentral acute middle maculopathy secondary to central retinal vein occlusion. Eye.

[34] Bhavsar KV (2016). Acute macular neuroretinopathy: A comprehensive review of the literature. Surv. Ophthalmol..

[35] McLeod D (2005). Why cotton wool spots should not be regarded as retinal nerve fibre layer infarcts. Br. J. Ophthalmol..

[36] de Carlo TE, Romano A, Waheed NK, Duker JS (2015). A review of optical coherence tomography angiography (OCTA). Int. J. Retin. Vitr..

