# Supplementary material for: Cerebral malaria: insight into pathology from optical coherence tomography
Source: Sci Rep. 2021 Aug 3;11:15722. doi: 10.1038/s41598-021-94495-9 (PMC8333417; doi:10.1038/s41598-021-94495-9)
Supplement: Supplementary file 1 — Supplementary Information. [file 41598_2021_94495_MOESM1_ESM.pdf]

Supplementary Data

Cerebral Malaria: Insight into Pathology from Optical Coherence Tomography

Zhanhan Tu (MBChB, MM, Ph.D.), Jack Gormley (MBChB), Viral Sheth (Ph.D.), Karl B. Seydel (M.D., Ph.D.), Terrie Taylor (D.O.), Nicholas Beare (FRCOphth), Valentina Barrera (Ph.D.), Frank A. Proudlock (Ph.D.), Chatonda Manda (MBChB), Simon Harding (FRCOphth), Irene Gottlob (MD. Univ Doz, FRCOphth)

Supplementary Table S1: Blantyre Coma Score 2
Supplementary Table S2: Demographics and diagnoses of comatose patients without cerebral malaria 3
Supplementary Table S3: OCT characteristics of CM patients with MR on admission 4

Supplementary Figure S 1: Macular fundus photography and fluorescein angiography of control child (48 months female) without retinopathy 5
Supplementary Figure S 2: Comparisons of hyper-reflective capillaries and vessels in patients with MR and age- and gender-matched healthy controls 6
Supplementary Figure S 3: Hemorrhage and cystoid macular edema (CME) 7
Supplementary Figure S 4: Comparisons of thicknesses of retinal layers between admission and CM follow-up 9
Supplementary Figure S 5: Comparisons of hyper-reflective foci in patients with diabetic macular edema and hyper-reflective capillaries in patients with MR 10
Supplementary Figure S 6: Comparisons of paracentral acute middle maculopathy (PAMM) and HRAs in patients with MR 11

Reference 12

Supplementary Table S1: Blantyre Coma Score

The Blantyre Coma Score is the sum of best motor response score, verbal response score and eye movement score. The minimum score is 0 (poor), the maximum score is 5 (good); scores less than 4 are abnormal.

Blantyre Coma Score for Children	
**Best motor response**	**Score**
Localizes painful stimulus	2
Withdraws limb from pain	1
Non-specific or absent response	0
**Verbal response**	**Score**
Appropriate cry	2
Moan or inappropriate cry	1
None	0
**Eye movement**	**Score**
Directed	1
Not directed	0

Supplementary Table S2: Demographics and diagnoses of comatose patients without cerebral malaria

F= female; M= Male.

ID	Gender	Age (month)	Diagnosis
1	F	19	Septic Shock
2	M	11	Meningitis
3	M	90	Acute bacterial meningitis
4	F	19	Sepsis, Meningitis and Malnutrition
5	F	121	Meningitis and Severe Immunosuppression
6	M	68	Tuberculosis and Meningitis

Supplementary Table S3: OCT characteristics of CM patients with MR on admission

Characteristics in OCT	Affected eye numbers	Percentage of eyes
**Hyper-reflective capillaries**	80	93%
**Hyper-reflective vessels**	78	90%
**Hyper-reflective areas**	70	81%
**Cotton wool spot**	32	37%
**Hemorrhage**	24	28%
**Cystoid macular edema**	8	9%

Supplementary Figure S 1: Macular fundus photography and fluorescein angiography of control child (48 months female) without retinopathy

A. En-face: a near infrared light OCT image of the retina which contains 80 B-scans; B. B-scan contains 600 A scans corresponding to the yellow line shown on the en-face image in the centre of the fovea (A). C. Macula OCT b-scan analysis. Measurement of thicknesses of inner and outer retinal layers centrally and 4 to 6° from the central fovea temporarily and nasally. Colored lines were placed semi-automatically at the interface of retinal layers to measure the thickness. Inner retinal layers (from inner limiting membrane to anterior surface of the outer plexiform layer) include retinal nerve fibre layer (RNFL); ganglion cell layer and inner plexiform layer complex (GCL-IPL); inner nuclear layer (INL); outer plexiform layer (OPL). Outer retinal layers (from anterior surface of the outer plexiform layer to cone outer segment tips) include outer nuclear layer (ONL); inner segment of photoreceptors (IS); outer segment of photoreceptors (OS); retinal pigment epithelium (RPE). D. Fundus photo and E. Fluorescein angiography without retinopathy.

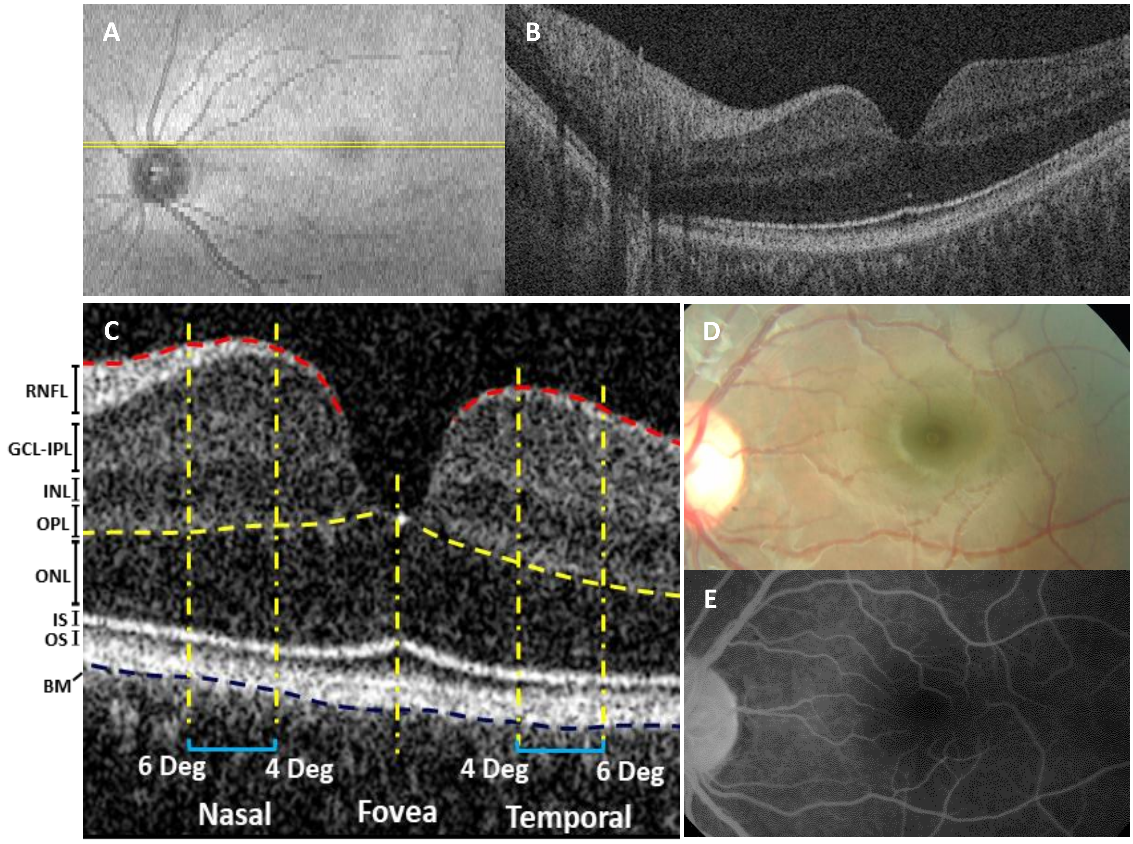

Supplementary Figure S 2: Comparisons of hyper-reflective capillaries and vessels in patients with MR and age- and gender-matched healthy controls

A. OCT B scan: Hyper-reflective dots (blue arrows) found in the inner retinal layers in a 7-month-old male patient with MR. B. OCT B-scan in a similar location of the retina from a 6-month-old male healthy participant. C. OCT B-scan shows hyper-reflective oval structures (yellow arrows) in the inner retinal layers of an 84-month-old female participant. D. OCT B-scan in a similar location in a healthy 85-month-old female control without abnormal hyper-reflective oval structures.

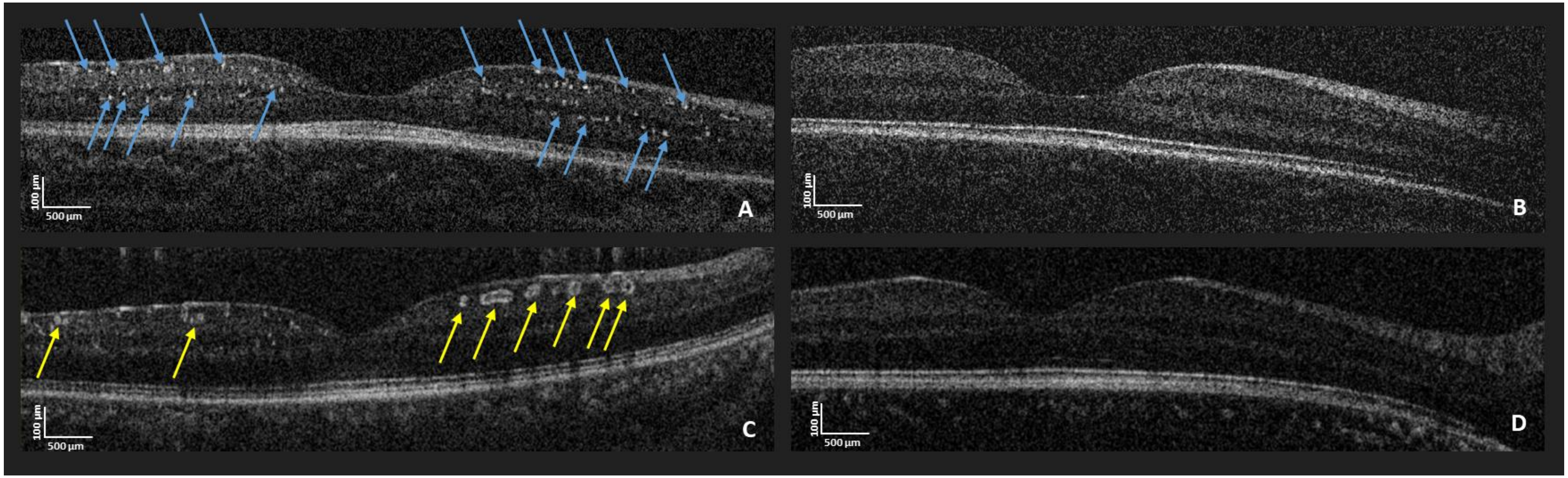

Supplementary Figure S 3: Hemorrhage and cystoid macular edema (CME)

S3.1.A Superficial (red arrow) and deep (yellow arrow) hyper-reflective lesion distorting underlying retinal layers on OCT B-scan in left eye of a 12-month-old female with CM at admission. B. En-face OCT showing superficial (red arrow) and deep (yellow arrows) round dark lesions corresponding to pathological changes in figure A; the fine yellow line corresponds to the left B-scan in A. C. Representative histology of superficial retinal hemorrhage (black arrow) and deeper hemorrhage (black arrowhead) from a different MR patient who died at Queen Elizabeth Central Hospital between 1996 and 2010. D. Fundus fluorescein angiography and E. fundus photo corresponding to OCT in A showing that the lesions from patient in A correspond to a new (red arrow) and an old hemorrhages (yellow arrow) masking underlying vessels and capillaries on fluorescein angiography. The dashed square corresponds to en-face OCT in B. The red dashed line corresponds B-scan in A (red arrow) and the yellow dashed line corresponds to B-scan in A (yellow arrow). Red and yellow arrows correspond to the same fundus locations in A, B, D and E.

S3.2.A. OCT B-scan of CME (blue arrow showing fluid in the outer nuclear layer and yellow arrow subretinal fluid) in the left eye of a 42-month old male CM patient at admission; B. En-face OCT; fine yellow line: location of the B-scan in A; blue and yellow arrows correspond to blue and yellow arrows in A, there is an extended dark area corresponding to retinal fluid; C. Representative histology of section through the fovea and macula demonstrating pseudophakic CMO, which is not from a patient with CM. Cyst-like spaces are present in the outer plexiform layer (Black arrow). (Image courtesy of Ralph C. Eagle, Jr., M.D., Philadelphia, PA. Pathologic Correlates in Ophthalmoscopy); D. Corresponding fluorescein angiography and E. fundus photo from patient in A. The dashed square, the dashed line and blue and yellow arrows correspond to en-face OCT in B and B-scan in A respectively. Blue and yellow arrows correspond to the same fundus locations in A, B, D and E. CMO was not detected on fundus photo and fluorescein angiography.

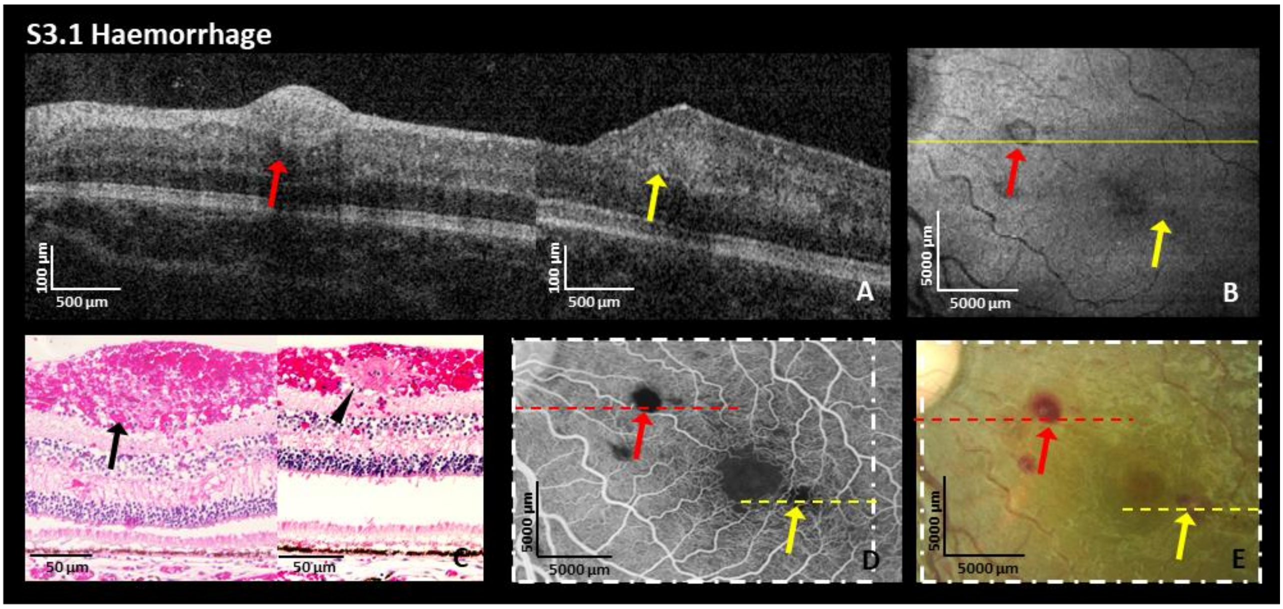
S3.1 Haemorrhage
100 µm
500 µm
100 µm
500 µm
A
5000 µm
5000 µm
B
50 µm
50 µm
C
5000 µm
5000 µm
D
5000 µm
5000 µm
E

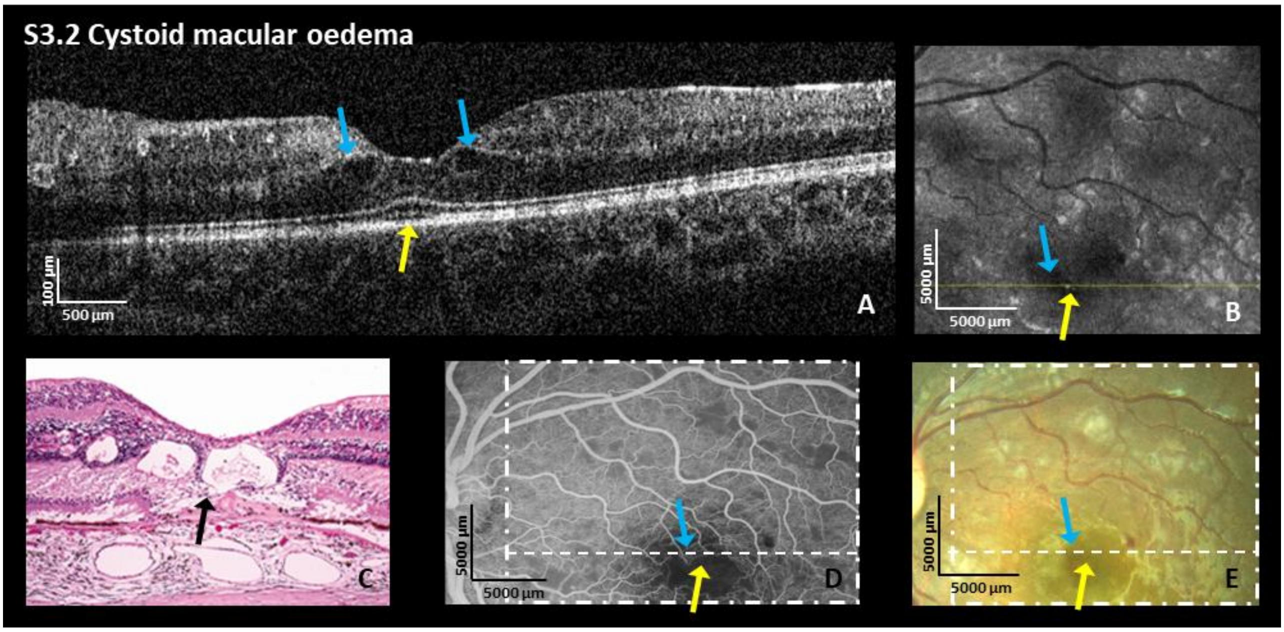
S3.2 Cystoid macular oedema
100 µm
500 µm
A
5000 µm
5000 µm
B
C
5000 µm
5000 µm
D
5000 µm
5000 µm
E

Supplementary Figure S 4: Comparisons of thicknesses of retinal layers between admission and CM follow-up

A: Comparison of thickness of inner retinal layers between admission and CM patients at one-month follow-up. B: Comparisons of thicknesses of inner retinal layers between admission and CM patients at one-year follow-up. Error bars in boxplot are the ranges of data. *: p <0.05, ***: p <0.001 and ****: p <0.0001 show significant different between two groups. Control groups are in green and patient groups in blue (Adm= admission; CM= cerebral malaria; 1M= one-month follow-up; 1Y= one-year follow-up).

A. Comparison of retinal layers between admission and 1-month follow-up in CM patients

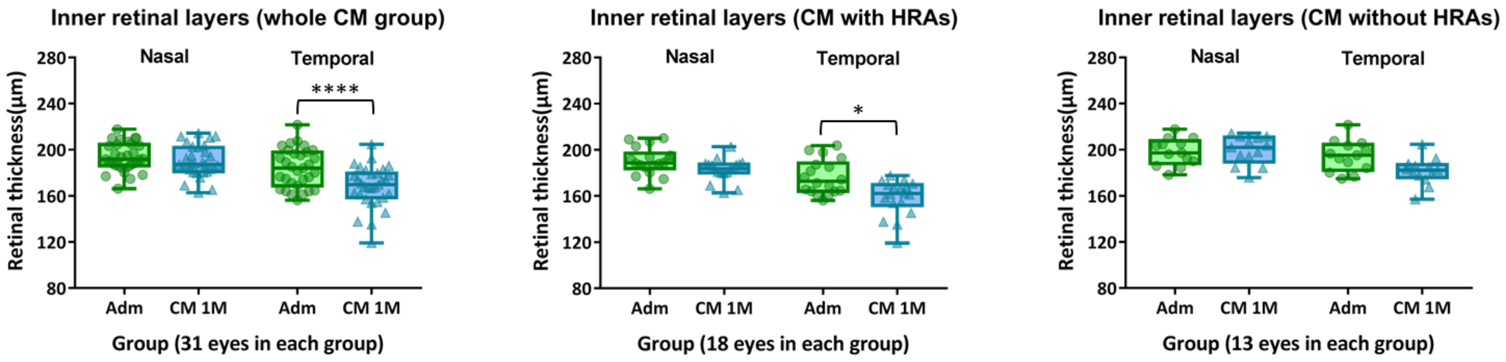

B. Comparison of retinal layers between admission and 1-year follow-up in CM patients

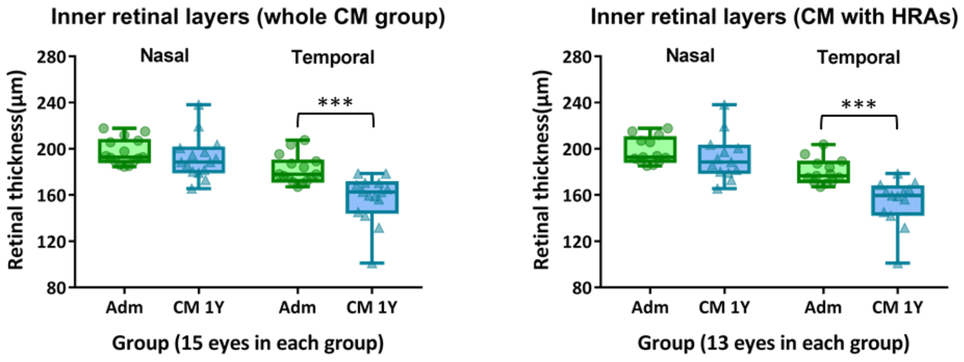

Supplementary Figure S 5: Comparisons of hyper-reflective foci in patients with diabetic macular edema and hyper-reflective capillaries in patients with MR

A: OCT B-scan of the right eye from a 76-year-old female with diabetic macular edema. (Image: courtesy of Mr James Deane, University Hospitals of Leicester, Leicester UK). The hyper-reflective foci (blue arrows) are present throughout all the layers of the retina. B: OCT B-scan of the right eye from a 7-months-old female with MR. The hyper-reflective dots (yellow arrows) are only limited within the inner retinal layers and are co-localized to retinal capillary plexuses.

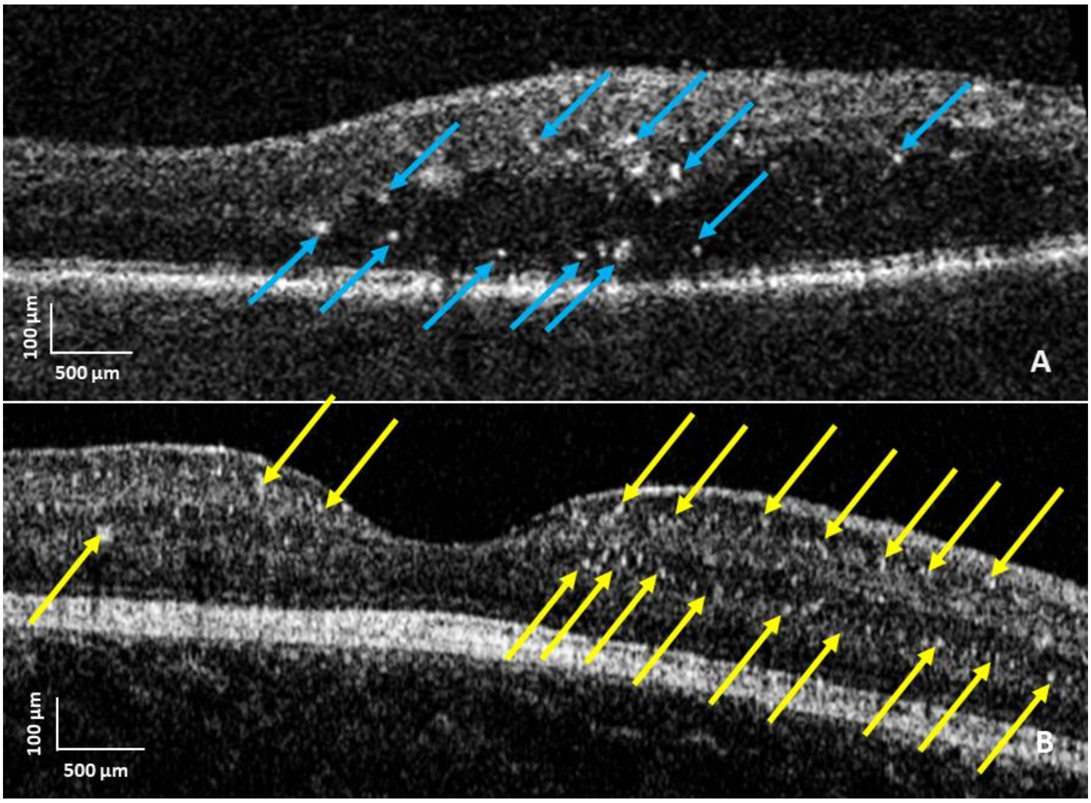

Supplementary Figure S 6: Comparisons of paracentral acute middle maculopathy (PAMM) and HRAs in patients with MR

A: OCT B-scan from a patient with PAMM (Reprinted with permission from Sally Chu, Peter L. Nesper, *et al*. Projection-Resolved OCT Angiography of Microvascular Changes in Paracentral Acute Middle Maculopathy and Acute Macular Neuroretinopathy. Invest. Ophthalmol. Vis. Sci. 2018;59(7):2913-2922.) [1] Hyper-reflective areas are (yellow circles) are features of PAMM at the interface of the inner nuclear layer (INL), outer plexiform layer (OPL) and outer nuclear layer (ONL). B: OCT B-scan from a patient with MR-positive. Yellow circles are showing hyper-reflective areas (HARs) located in the same layers and similar distribution as in PAMM in image A.

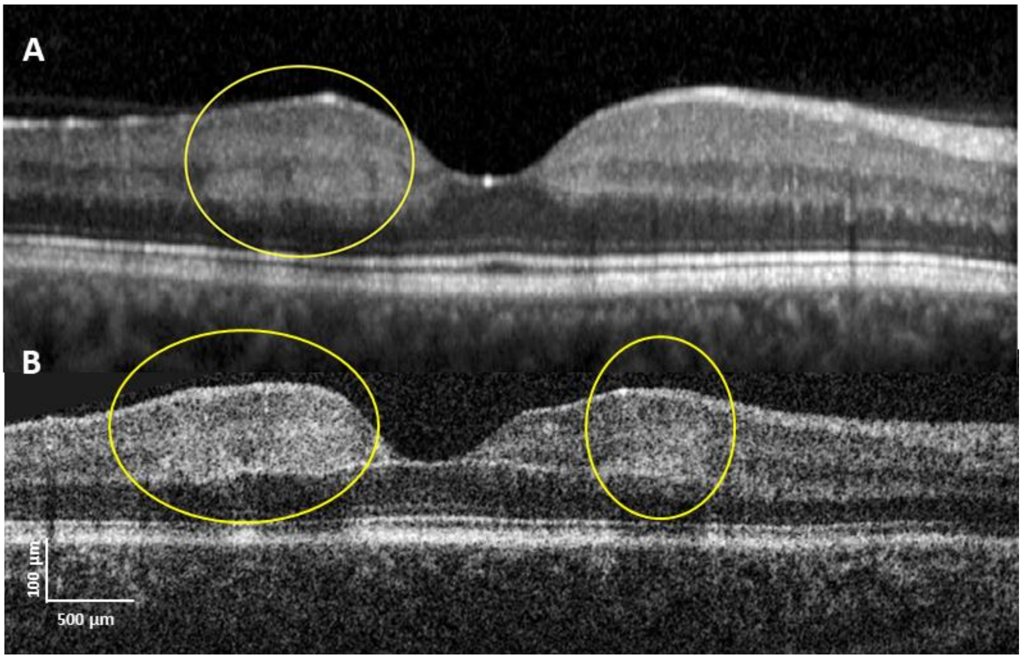

Reference

[1] Chu S, Nesper PL, Soetikno BT, Bakri SJ, Fawzi AA. Projection-Resolved OCT Angiography of Microvascular Changes in Paracentral Acute Middle Maculopathy and Acute Macular Neuroretinopathy. Investigative Ophthalmology & Visual Science 2018;59(7):2913-22.
